# Can Gut Microbiota Be a Good Predictor for Parkinson’s Disease? A Machine Learning Approach

**DOI:** 10.3390/brainsci10040242

**Published:** 2020-04-19

**Authors:** Daniele Pietrucci, Adelaide Teofani, Valeria Unida, Rocco Cerroni, Silvia Biocca, Alessandro Stefani, Alessandro Desideri

**Affiliations:** 1 Department of Biology, University of Rome Tor Vergata, 00133 Rome, Italy; daniele.pietrucci@uniroma2.it (D.P.); Adelaide.Teofani@uniroma2.it (A.T.); valeria.unida@gmail.com (V.U.); 2 UOSD Parkinson’s Center, Department of Systems Medicine, University of Rome Tor Vergata, 00133 Rome, Italy; rocco.cerroni@gmail.com (R.C.); Stefani@uniroma2.it (A.S.); 3 Department of Systems Medicine, University of Rome Tor Vergata, 00133 Rome, Italy; biocca@med.uniroma2.it

**Keywords:** Parkinson’s disease, gut microbiota, machine learning, predictor, gut–brain axis

## Abstract

The involvement of the gut microbiota in Parkinson’s disease (PD), investigated in several studies, identified some common alterations of the microbial community, such as a decrease in *Lachnospiraceae* and an increase in *Verrucomicrobiaceae* families in PD patients. However, the results of other bacterial families are often contradictory. Machine learning is a promising tool for building predictive models for the classification of biological data, such as those produced in metagenomic studies. We tested three different machine learning algorithms (random forest, neural networks and support vector machines), analyzing 846 metagenomic samples (472 from PD patients and 374 from healthy controls), including our published data and those downloaded from public databases. Prediction performance was evaluated by the area under curve, accuracy, precision, recall and F-score metrics. The random forest algorithm provided the best results. Bacterial families were sorted according to their importance in the classification, and a subset of 22 families has been identified for the prediction of patient status. Although the results are promising, it is necessary to train the algorithm with a larger number of samples in order to increase the accuracy of the procedure.

## 1. Introduction

Neurodegenerative diseases represent a heterogeneous class of neurological disorders, with a high social, sanitary and economic impact. Parkinson’s disease (PD) is one of the most common neurodegenerative diseases, with a worldwide prevalence of 0.4% that is likely to double in the next 20 years [1]. The PD phenotype is characterized by movement disorders as a result of the loss of dopaminergic neurons in the substantia nigra caused by α-synuclein (α-syn) aggregates [2]. Only 10% of PD cases are due to genetic causes [3], indicating that environmental factors like dietary habits, head injury and nicotine consumption could trigger or influence the progression of PD [4]. Among environmental factors, the role of gut microbiota and its interactions with the gut–brain axis aroused the interest of researchers worldwide [5]. Gut microbiota can interact with the vagus nerve using neuroimmune and neuroendocrine mechanisms and, at the same time, the nervous system can modulate the gut physiology and environment, affecting the gut microbiota composition [6]. PD is influenced by this bidirectional communication, and PD patients show significant comorbidity with small-intestine bacterial overgrowth, constipation and Irritable Bowel Disease (IBD) like symptoms [7]. Furthermore, α-syn aggregates are found in the enteric nervous system and can spread to the central nervous system through the vagus nerve, and gut microbiota can affect the aggregation of α-syn [8]. 

The role of gut microbiota was evaluated in PD patients in several studies, mainly using targeted metagenomics and sequencing the 16S rRNA gene from fecal samples [2,9,10,11,12,13,14,15,16,17]. Some common features have been found, such as the reduction of bacteria producing short-chain fatty acids from the *Lachnospiraceae* family [5]. Regarding other bacterial families, the results are contradictory; for example, the *Bifidobacteriaceae* family was reported to have a lower abundance in PD patients in some studies [2,18] and higher abundances in others [11,19]. So, although all studies indicate a correlation between microbiota and Parkinson’s disease, there is no convergence as of yet on the bacterial families identifiable as specific biomarkers. To overcome this problem, we analyzed all published data with a computational procedure that can identify taxa involved in the pathology. Machine learning (ML) algorithms are appropriate tools to create predictive models that can distinguish the pathological status of a patient using the frequencies of bacteria in the feces. 

ML algorithms are currently used for building predictive models for the classification of biological data, and identify biomarkers through a training procedure [20,21]. This technology was applied to identify marker genes in breast cancer [22], and to analyze clinical data for predicting cardiovascular and diabetes risk [23,24]. Recently, ML algorithms have been used to identify biomarkers analyzing shotgun and 16S rRNA data [25].

In this study, we use three different supervised ML algorithms to analyze 16S rRNA gene sequencing data derived from six different studies [2,9,10,11,12,13] downloaded from the Sequence Read Archive database. From this analysis, we obtain a classifier that can predict the pathological status of PD patients against healthy controls (HCs), and we identify a subset of 22 bacterial families that are discriminative for the prediction. 

## 2. Materials and Methods

### 2.1. Datasets Downloadand Bioinformatic and Statistical Data Analysis

Datasets were downloaded from the Sequencing Read Archive (SRA) database [26] using the SRA Toolkit (http://ncbi.github.io/sra-tools/). Raw reads from the following BioProject IDs were selected: PRJNA510730 [9], PRJNA268515 [2], PRJEB14674 [13], PRJEB14928 [11], PRJNA381395 [12] and PRJEB27564 [10]. Non-fecal samples from PRJNA268515 and PRJNA381395 studies were removed before the analysis. The quality of raw sequencing reads was assessed with FastqC (https://www.bioinformatics.babraham.ac.uk/projects/fastqc/), and the bioinformatic data analysis was performed using the QIIME 2 pipeline [27]. Reads were quality filtered, chimera-checked and clustered in amplicon sequencing variants (ASVs) using DADA2 in the QIIME 2 pipeline [28]. The taxonomy of representative sequences was assessed using the q2-feature-classifier and the GreenGenes database [29,30]. Data normalization and filtering were performed using R 3.5.3. The dataset was loaded in R using the phyloseq package (version 1.26.1) [31]. Clusters (ASVs) with a number of reads lower than 0.05% of the total read number were removed from the analysis [32]. The read number count was normalized using DESeq2 [33]. The normalized table was summed up at the Family level using the tax_glom function in phyloseq.

### 2.2. Machine Learning Data Analysis

ML data analysis was performed using custom scripts in Python 3.6.7 language, using the sci-kit learn package (https://scikit-learn.org/stable/). Bacterial families were normalized across all samples using the StandardScaler method, which scales the distribution by subtracting the mean from each value and dividing the difference by the standard deviation. We used this type of standardization since, in the microbiota, some bacterial families are more abundant than others and their abundances can widely vary across samples.

The performance was evaluated using a stratified cross-validation (CV) with a K value equal to 5. The dataset was divided into training and test sets 5 times, allowing the training and the testing of the algorithms in 5 different iterations (folds). Each time, a different portion of the dataset was used in the training or the test set, in order to predict all the samples. In this way, we reduced the bias of the random division of samples in the training or test set. 

We compared the performances of three different machine learning algorithms: (1) a random forest classifier (RF) [34], with 2000 estimators (trees) and a depth of 66 nodes (leaves); (2) a neural network (NN) classifier [35], with 3 hidden layers and respectively 180, 90 and 45 neurons for each layer; and (3) a support vector machine (SVM) classifier [36], with a polynomial kernel and cost parameter equal to 1.

The performance was evaluated using true positive cases (TP), false positive cases (FP), true negative cases (TN) and false negative cases (FN). A TP is a PD patient correctly classified as PD patient, while an FP is a PD patient incorrectly classified as HC. Conversely, a TN is an HC correctly classified as HC and an FP is an HC incorrectly classified as PD patient.

For each algorithm, at each fold, the number of TP, FP, TN and FN cases was computed and used to summarize the following metrics: accuracy, precision, recall, F-score and area under the curve (AUC). The accuracy is defined as the ratio of correctly predicted observations; the recall (or true positive rate, TPR) is the proportion of actual positives that are correctly identified as positive; the precision is the ratio of positive classifications identified correctly; and the F-score is the harmonic mean between precision and recall. The AUC was computed using the receiver operating characteristic (ROC) curve. The ROC curve summarizes the true positive rate and the false positive rate, and the AUC indicates the ability of the classifier to distinguish between two classes (i.e., PD or HC).

The importance of each bacterial family in the RF algorithm was evaluated using the “embedded feature selection strategy’’ analyzing the “Gini impurity decrease” [21]. The bacterial families were sorted from the most to the least relevant. The RF algorithm was then re-trained systematically using the first ‘’n’’ bacterial families, starting from the first n = 5 families and increasing this number until the AUC, the precision, the recall and the F-score values were comparable to those obtained with the whole family set (n = 52).

## 3. Results

### 3.1. Datasets Description and Supervised Machine Learning Approach

A total of 873 16S rRNA gene sequencing data of fecal samples from PD patients and HCs were downloaded from the Sequence Read Archive (SRA) database to study the association between the microbiota dysbiosis and diagnosis of PD. The distribution of PD and HC samples and the methodological approaches are reported in Table 1. 

After the bioinformatic analysis, 846 samples were retained. PD patients (472 samples) represent 56% of the whole dataset. We uniformly processed the metagenomic data of all samples, using supervised ML algorithms. In detail, random forest (RF), neural network (NN) and support vector machine (SVM) were used for this evaluation since they are state-of-the-art approaches and are appropriate for this type of data [37]. The dataset was analyzed using cross-validation, randomly selecting 80% of the samples to create the training set, and the remaining 20% to create the test set and evaluate the prediction. The model was evaluated by resampling the test and training set 5 times, using a stratified cross-validation (K-fold = 5).

**Figure 1:**
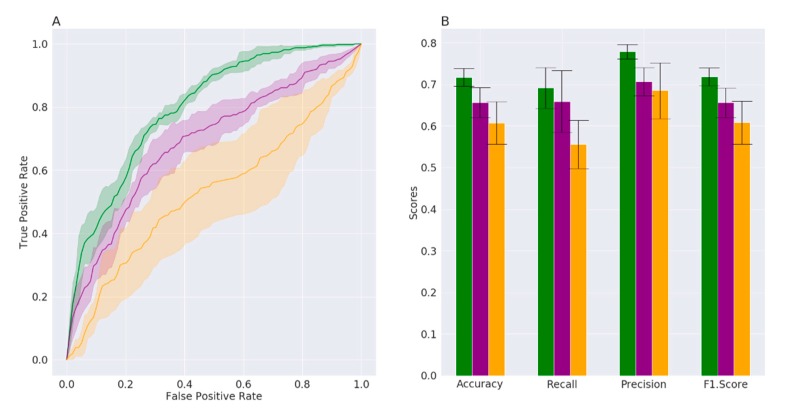
(**A**) Average ROC curves (over 5 folds) with confidence intervals and (**B**) prediction performance metrics with the relative margin of error. The results for random forests are reported in green, for neural networks in purple and for support vector machines in orange, respectively.

For each approach, the algorithm parameters were tuned through a grid search and were selected to optimize the training phase. Prediction performance was evaluated by the AUC metrics, which summarize true-positive and false-positive rates. The comparison of the three methods is reported in Figure 1A,B. The AUC is significantly higher for RF (0.80 ± 0.01) than for NN (0.67 ± 0.03) and SVM (0.54 ± 0.08) (Figure 1), indicating that RF is the most effective algorithm in distinguishing between status (PD or HC), according to the bacterial families’ frequencies in the feces. In line with this, Figure 1B indicates that the accuracy, precision, recall and F-score are higher for RF than for NN and SVM. 

### 3.2. Feature Selection

Overall, the RF algorithm showed the best performance and has been selected to rank the importance of the bacterial families for the prediction. This process, defined as “feature selection”, identifies the most informative and relevant features in the classification of the PD status. The ranking of the bacterial families in discriminating between HC and PD patients is reported in Table 2.

In order to identify the minimal number of bacterial families that can reliably predict the pathological status, an embedded feature selection strategy was performed by re-training the RF algorithm with a subset of families and comparing the corresponding metrics. Initially, the subset included only the first 5 families in the ranking, shown in Table 2. The number of families was systematically increased to re-train the algorithm until the AUC, the precision, the recall and the F-score values were close enough to those obtained using the 52 total number of families for the training. This process allowed for the removal of bacterial families not strictly involved in the gut dysbiosis of Parkinson’s disease. The results indicate that by training the model with the first 22 bacterial families, the AUC, precision, recall the F-score values were almost identical to those obtained using the total number of families (Table S1), suggesting that this is the minimal subset that can be considered to correctly predict the pathological status.

**Figure 2:**
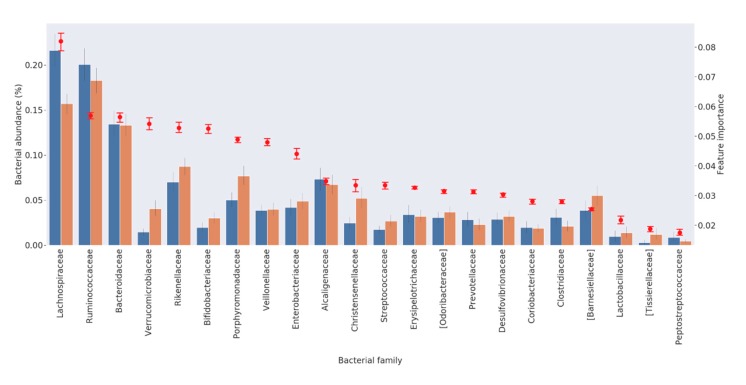
List of the 22 bacterial families required for discriminating between HC and PD patients. For each family, the average percentage of abundance is represented by a bar, orange for HC and blue for PD patients (left scale). The importance of the family in discriminating the status is represented by a red dot (right scale).

The importance of the first 22 families in discriminating between patients and controls is plotted in Figure 2, together with their relative abundance. Interestingly, the plot shows that the importance of each family is not directly correlated with the relative family abundance in the samples. In some cases, we detected relevant species with low prevalence but high discriminative potential between “healthy” and “diseased” subjects. For example, *Verrucomicrobiaceae/Akkermansiaceae* and *Bifidobacteriaceae* were highly discriminative, although with a low average relative abundance.

It is also worth noting that not all of the 22 families identified in this analysis are cited in the previous studies investigating the role of gut microbiota in PD dysbiosis. In fact, among the first ten families identified as the most important ones in the PD diagnosis, eight were already identified in the literature, but two families—namely, *Veillonellaceae* and *Alcaligenaceae*—have never been reported before (Table 2). 

## 4. Discussion

In this study we present, for the first time, an ML data analysis on microbiota dysbiosis in PD patients. We considered six available datasets from the SRA database, obtained from experiments carried out in different laboratories (Table 1). We downloaded and re-analyzed the datasets, uniformly processing the data using the most up-to-date bioinformatic procedures. 

We initially evaluated the efficiency of three ML algorithms (RF, SVM and NN) in identifying samples belonging to HC or PD patients comparing different metrics (AUC, accuracy, precision, recall and F-score). The RF algorithm exhibited the best results providing an AUC of 80% and accuracy of 71%. This result is satisfying since we are analyzing data from studies that differ for participants’ nationality and for several methodological aspects, such as DNA extraction kit, sample transport and conservation (Table 1).

It is worth noting that, by analyzing the microbiota of diseases directly located in the gut, higher AUC values have been reported [23]. On the other hand, AUC values around 80% are in line with studies on the prediction of pathologies not directly related to the gut, such as obesity using 16S rRNA metagenomic data [25], or type-2 diabetes using shotgun metagenomic data [23]. This might indicate that the AUC value has reached the limit for Parkinson’s disease, although we cannot exclude that this value could be improved by increasing the sample size. 

The relatively low accuracy (71%) could be due to various methodological approaches used in different laboratories in collecting, storing and processing data. As critically pointed out in a recent review [5], methodological inconsistencies between gut microbiome case–control studies in PD might contribute to the heterogeneity of the results. A lack of unique experimental and bioinformatic protocols prevents a direct and straightforward comparison of the data. We emphasize the importance of defining unique standards to permit a reliable comparison.

We found a subset of 22 bacterial families that provide prediction metrics almost identical to those obtained when the RF algorithm was trained with the whole microbiota (52 families). Subsets made by a lower number of relevant bacterial families (i.e., 5, 10) did not provide similar results, indicating that the combination of fewer species is insufficient to characterize the microbiota associated with this disease (Supplementary Table S1). This finding indicates the presence of a complex interplay of numerous bacterial families involved in gut dysbiosis in Parkinson’s disease. Interestingly, the rank of importance of each bacterial family is not directly correlated to its relative abundance.

Not all families identified by the RF algorithm were reported in the literature (Table 2). Indeed, eight of the first ten families in the rank (*Lachnospiraceae*, *Ruminococcaceae*, *Bacteroidaceae*, *Verrucomicrobiaceae*/*Akkermansiaceae*, *Rikenellaceae*, *Bifidobacteriaceae*, *Porphyromonadaceae*, *Enterobacteriaceae*) are cited, whilst two families, *Veillonellaceae* and *Alcaligenaceae*—both higher in PD patients—have never been reported before. The identification of new bacterial families that may play an important role in predicting the PD status highlights the power of a prediction analysis based on ML algorithms.

Finally, we want to point out that the values of the predictive metrics are probably too low for an immediate application of the procedure for the purpose of Parkinson’s disease’s diagnostics; however, the ranking of importance of bacterial families involved in the disease may help in its diagnosis.

## 5. Conclusions

In this work, we processed 846 16S rRNA microbiota data coming from six different studies, applying an ML approach. The RF algorithm provided an AUC of 80% and accuracy of 71% and identified a subset of 22 families that can be used to discriminate between PD and HC.

Unfortunately, the data deposited in the public databases are only a small fraction of the data that has been published up to now. This has prevented us from analyzing a larger number of data and increasing the population of the training and test sets. It is necessary to train the RF algorithm with a higher number of samples in order to increase the accuracy of the model and provide more robust results on the association between the gut microbiota and PD. We propose that the scientific community should build a network to share all the data produced by different laboratories, permitting the development of a fully reliable tool for the diagnosis and prognosis of this disease.

## Figures and Tables

**Table 1:** List of references, number of samples, methodological approaches and nationality of studies considered in this analysis.

Reference	PD Samples	HC Samples	Sample Transport	DNA Extraction	16S Region	Nationality
				Method		
[2]	34	31	BD Gaspak	FastDNA Spin Kit for Soil	V4	United States
[10]	65	68	NR	PSP Spin Stool Kit	V3-V4	Finland
[9]	116	82	Stabilizer PSP	PSP Spin Stool Kit	V3-V4	Italy
[13]	206	133	Ambient temp	Earth microbiome project protocol	V4	United States
[12]	22	34	Stabilizer PSP	PSP Spin Stool Kit	V3-V4	Germany
[11]	29	26	Immediate freezing	Custom Protocol Hopfner	V4	Russia

**Table 2:** Ranking of the importance of the bacterial families in discriminating between healthy controls and Parkinson’s disease (PD) patients.

Bacterial Family	Ranking of Importance	Higher (−) or Lower (+) Abundance in PD Patients from RF Algorithm	References in the Literature Reporting Overabundance in PD Patients	References in the Literature Reporting Lower Abundance in PD Patients
***Lachnospiraceae***	1	−	[14]	[2,9,10,11,13,15,16,17]
***Ruminococcaceae***	2	−	[2,14]	[13,15]
***Bacteroidaceae***	3	−	[2]	[16]
***Verrucomicrobiaceae***	4	+	[2,12,13,17]	
***Rikenellaceae***	5	+	[19]	
***Bifidobacteriaceae***	6	+	[13,16,17,19]	[2,18]
***Porphyromonadaceae***	7	+	[17]	
***Veillonellaceae***	8	+		
***Enterobacteriaceae***	9	+	[9,15,17]	
***Alcaligenaceae***	10	−		
***Streptococcaceae***	11	+		[19]
***Christensenellaceae***	12	+	[13,16,17]	
***Erysipelotrichaceae***	13	+	[19]	
***[Odoribacteraceae]***	14	+		
***Prevotellaceae***	15	−		[10,16,17]
***Desulfovibrionaceae***	16	+	[14,19]	
***Coriobacteriaceae***	17	−	[17]	
***Clostridiaceae***	18	−	[2,10,16]	[19]
***[Barnesiellaceae]***	19	+		
***Lactobacillaceae***	20	+	[9,10,11,16]	
***[Tissierellaceae]***	21	+		
***Peptostreptococcaceae***	22	−	[16,17]	[14,15]
***Methanobacteriaceae***	23	+		[19]
***[Mogibacteriaceae]***	24	−		
***[Paraprevotellaceae]***	25	+		
***Turicibacteraceae***	26	−		
***Pseudomonadaceae***	27	+		
***Victivallaceae***	28	−		
***Campylobacteraceae***	29	+		
***Synergistaceae***	30	+		
***Pasteurellaceae***	31	−	[14]	[10,13,19]
***Corynebacteriaceae***	32	+		
***S24-7***	33	−		
***Enterococcaceae***	34	+	[9,11,15,17]	
***Actinomycetaceae***	35	+		
***Moraxellaceae***	36	−		
***Burkholderiaceae***	37	−		
***Comamonadaceae***	38	+		
***Alcanivoracaceae***	39	−		
***Oxalobacteraceae***	40	−		
***Propionibacteriaceae***	41	−		
***Xanthomonadaceae***	42	−		
***Rhodobacteraceae***	43	−		
***Fusobacteriaceae***	44	+		
***Staphylococcaceae***	45	−		
***Caulobacteraceae***	46	+		
***Caldicoprobacteraceae***	47	−		
***Succinivibrionaceae***	48	+		
***Peptococcaceae***	49	−		
***Flavobacteriaceae***	50	+		
***[Weeksellaceae]***	51	+		
***Aeromonadaceae***	52	+		

## Supplementary Materials

The following are available online at https://www.mdpi.com/2076-3425/10/4/242/s1. Table S1: Random forest performance with a reduced number of bacterial families.
