# Supplementary material for: Can Gut Microbiota Be a Good Predictor for Parkinson’s Disease? A Machine Learning Approach
Source: Brain Sci. 2020 Apr 19;10(4):242. doi: 10.3390/brainsci10040242 (PMC7226159; doi:10.3390/brainsci10040242)
Supplement: Supplementary file 1 [file brainsci-10-00242-s001.pdf]

Article Title: Can gut microbiota be a good predictor for Parkinson's Disease? A machine learning approach.

Daniele Pietrucci[1+], Adelaide Teofani[1+], Valeria Unida[1], Rocco Cerroni[2], Silvia Biocca[3], Alessandro Stefani[2], Alessandro Desideri*[1]

[+]These authors have contributed equally to this work.

Authors' information

[1]Department of Biology, University of Rome Tor Vergata, Rome, Italy

[2]UOSD Parkinson's Center, Department of Systems Medicine, University of Rome Tor Vergata, Rome, Italy

[3]Department of Systems Medicine, University of Rome Tor Vergata, Rome, Italy

Corresponding Author

*Alessandro Desideri, Department of Biology – Università Degli Studi di Roma Tor Vergata, Via della Ricerca Scientifica 1, 00133, Roma, Italy,desideri@uniroma2.it, +390672594376

Key-words: Parkinson's Disease, Gut Microbiota, Machine Learning, Predictor, Gut-Brain-Axis

Supplementary Tables

Table S1. Random Forest performance with a reduced number of bacterial families

Number of families	*AUC*	*Accuracy*	*Precision*	*Recall*	*F-Score*
**5**	0.74 ± 0.03	0.61 ± 0.08	0.73 ± 0.04	0.61 ± 0.07	0.6 ± 0.1
**10**	0.77 ± 0.01	0.64 ± 0.07	0.75 ± 0.03	0.62 ± 0.06	0.63 ± 0.09
**15**	0.78 ± 0.02	0.65 ± 0.06	0.76 ± 0.02	0.66 ± 0.05	0.65 ± 0.08
**20**	0.79 ± 0.02	0.66 ± 0.06	0.77 ± 0.02	0.67 ± 0.04	0.66 ± 0.07
**22**	0.8 ± 0.02	0.67 ± 0.06	0.78 ± 0.02	0.69 ± 0.04	0.66 ± 0.07
**52**	0.8 ± 0.02	0.71 ± 0.02	0.78 ± 0.02	0.69 ± 0.04	0.66 ± 0.07

The Random Forest algorithm was evaluated using a subset of bacterial families. The results using the most important 5, 10, 15, 20, 22 bacterial families, following the ranking of table2, are reported. Selection of the first 22 families provide results comparable to the selection of the full (52) families set.
